# Correlative Light and Electron Microscopy Reveals the HAS3-Induced Dorsal Plasma Membrane Ruffles

**DOI:** 10.1155/2015/769163

**Published:** 2015-09-10

**Authors:** Kirsi Rilla, Arto Koistinen

**Affiliations:** Institute of Biomedicine and SIB Labs, University of Eastern Finland, 70211 Kuopio, Finland

## Abstract

Hyaluronan is a linear sugar polymer synthesized by three isoforms of hyaluronan synthases (HAS1, 2, and 3) that forms a hydrated scaffold around cells and is an essential component of the extracellular matrix. The morphological changes of cells induced by active hyaluronan synthesis are well recognized but not studied in detail with high resolution before. We have previously found that overexpression of HAS3 induces growth of long plasma membrane protrusions that act as platforms for hyaluronan synthesis. The study of these thin and fragile protrusions is challenging, and they are difficult to preserve by fixation unless they are adherent to the substrate. Thus their structure and regulation are still partly unclear despite careful imaging with different microscopic methods in several cell types. In this study, correlative light and electron microscopy (CLEM) was utilized to correlate the GFP-HAS3 signal and the surface ultrastructure of cells in order to study in detail the morphological changes induced by HAS3 overexpression. Surprisingly, this method revealed that GFP-HAS3 not only localizes to ruffles but in fact induces dorsal ruffle formation. Dorsal ruffles regulate diverse cellular functions, such as motility, regulation of glucose metabolism, spreading, adhesion, and matrix degradation, the same functions driven by active hyaluronan synthesis.

## 1. Introduction

Hyaluronan is synthesized in the inner face of the plasma membrane by three isoforms of hyaluronan synthases (HAS1, 2, and 3), unique enzymes that simultaneously elongate, bind, and extrude the growing hyaluronan chain directly into extracellular space [1]. Active synthesis of hyaluronan enhances plasma membrane dynamics and formation of several types of actin-based plasma membrane protrusions, like filopodia [2], lamellipodia [3], and membrane ruffles [4].

Ruffles are flat plasma membrane folds that use the actin-based machinery for their dynamic reshaping [5]. Depending on source of studies, their nomenclature is variable, including dorsal ruffles, waves (because they resemble waves on a water surface), linear ruffles [6], or circular dorsal ruffles [7]. Two structurally similar but distinct types of ruffling have been reported, depending on their cellular location. Peripheral ruffling is typically associated with lamellipodia formation and migration [8], while dorsal ruffling is connected to macropinocytosis [9] and internalization of growth factor receptors [10].

Any cell types studied so far like keratinocytes [11], MCF-7 breast cancer cells, MDCK Kidney cells [12], chondrosarcoma cells, [13], fibroblasts, and mesothelial and melanoma cells [14, 15] are induced to grow special plasma membrane protrusions when overexpressing HAS2 or especially HAS3. Moreover, cell types with endogenously high hyaluronan secretion like fibroblasts [16, 17], smooth muscle cells, chondrosarcoma cells [13], neuroblastoma cells [18], and fibroblasts from Shar Pei dogs with high HAS2 expression [19] have high number of plasma membrane protrusions.

The studies of HAS-induced protrusions have been challenging because they are thin and fragile and difficult to preserve by fixation and other processing steps for light and electron microscopy [11–13]. Particularly protrusions arising from apical regions of the plasma membrane that are not adherent to the substratum are easily shrunk and collapsed. Thus their formation and maintenance of structure are still enigmatic and their functions are partly uncharacterized.

The aim of this work was to develop a simple, cost-effective method to correlate fluorescent signal from confocal laser scanning microscopy (CLSM) to fine morphology of the cells studied with scanning electron microscope (SEM). With this method, we confirmed the GFP-HAS3 localization into the plasma membrane and its protrusions, their bulbous tip complexes, as well as in plasma membrane ruffles. Surprisingly, it was found that in fact ruffle-like plasma membrane folds act as basis of HAS-induced protrusions, which has not been reported previously. Additionally, it was shown in detail for a first time that GFP-HAS3 not only localizes to dorsal ruffles but also induces dorsal ruffle formation. The results obtained in this work will bring us closer to the detailed characterization of hyaluronan-dependent plasma membrane protrusions, their regulation and functions. These structures are putative factors behind hyaluronan-driven effects in diseases like cancer, inflammation, and disorders of glucose metabolism.

## 2. Methods

### 2.1. Cell Culture

The human breast adenocarcinoma cell line, MCF-7, was cultured in minimum essential medium alpha (MEM*α*, EuroClone, Pavia, Italy) supplemented with 5% fetal bovine serum (FBS, HyClone, Thermo Scientific, Epsom, UK), 2 mM glutamine (EuroClone), 50 *μ*g/mL streptomycin sulfate, and 50 U/mL penicillin (EuroClone). Cells were passaged twice a week at a 1 : 5 split ratio using 0.05% trypsin (w/v) 0.02% EDTA (w/v) (Biochrom AG, Berlin, Germany).

### 2.2. Transfections

MCF-7 cells were grown on gridded glass bottom culture dishes (MatTek Corporation, Ashland, MA) before transient transfection with human HAS3 cDNA in-frame with an N-terminal GFP fusion protein [20]. The generation of stable doxycycline-inducible EGFP-HAS3 overexpressing MCF-7 cells was performed as described before [21]. For induction of GFP-HAS3 expression, 0.5 ng/mL of doxycycline (Sigma, St. Louis, MO, USA) was used.

### 2.3. Imaging, Image Processing, and Analysis

Next day, after transient transfection or induction of stable cells with doxycycline, the fluorescent images were obtained with Zeiss Axio Observer inverted microscope (10 × NA 1.3 or 63 × NA 1.4 oil objective) equipped with Zeiss LSM 700 confocal module (Carl Zeiss Microimaging GmbH, Jena, Germany) and an external DIC-capable transmitted-light channel. The cells were fixed with 2% glutaraldehyde either before or immediately after confocal imaging. To control the effect of hyaluronidase digestion on cell morphology, some samples were treated with* streptomyces* hyaluronidase (Seikagaku Kogyo Co., 5 TRU/mL, 30 min at 37°C) prior to fixation. Thereafter, the cells were routinely dehydrated in ascending series of ethanol and hexamethyldisilazane and finally coated with thin layer of gold. After processing, cells were imaged with Zeiss Sigma HD∣VP (Zeiss, Oberkochen, Germany) scanning electron microscope at 3 kV. Image processing, like 3-dimensional rendering, analysis of images, and further modification, was performed using ZEN 2012 software (Carl Zeiss Microimaging GmbH), ImageJ 1.32 software (http://rsb.info.nih.gov/ij/), and Adobe Photoshop 8.0.

SEM images (8-bit gray-level with pixel resolution of 1024 ∗ 728) were utilized to quantify the plasma membrane ruffling. From both groups, 20 cells were selected for analysis. Image analysis was carried out using ImageJ. A representative area from apical plasma membrane of the cells was outlined and thresholding was utilized to define ruffles from the background. After thresholding, the images were segmented so that the ruffles were separated with an automatic algorithm. Then the number and areas of discrete ruffles in each cell were calculated. The density of ruffles was presented as the number of ruffles over a cell area of 100 *μ*m^2^. It should be noticed that the SEM images were recorded using In-Lens detector which is a concentric detector inside the SEM column. The use of the In-Lens detector prevented shadowing effect which is typical to conventional secondary electron imaging.

### 2.4. Statistical Analysis

Statistical comparison was carried out using IBM SPSS Statistics software (ver. 19; SPSS Inc., Chicago, USA). Mann-Whitney *U* test was used to evaluate the difference in ruffling between the noninduced and induced cells. *P* values less than 0.05 were considered statistically significant.

## 3. Results

### 3.1. A simple Correlative Light and Electron Microscopy Method

**Figure 1:**
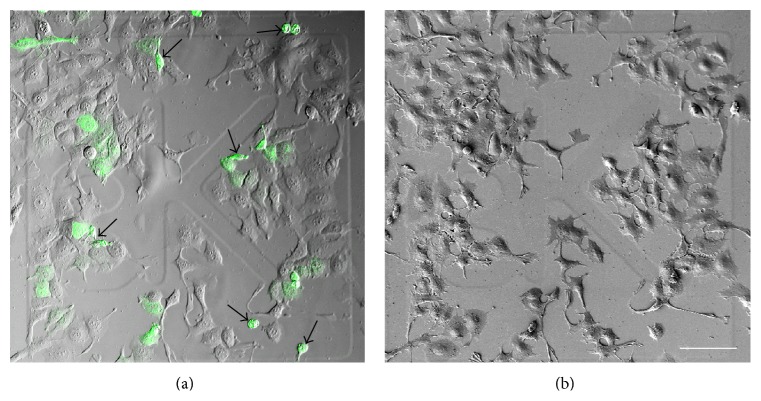
Utilization of gridded coverslips for localization of cultured cells for correlative imaging. Low magnification images showing an overview of transiently transfected cells imaged by CLSM and DIC (a) and the corresponding area imaged by SEM (b). Arrows in (a) indicate cells that were detached in the subsequent preparation steps for scanning electron microscopy. Scale bar 100 *μ*m.

This study presents a simple and easy process to image live or fixed cells by high resolution CLSM and SEM. The area imaged by CLSM was easily relocalized in SEM by utilizing gridded coverslips (Figure 1). A simultaneous DIC imaging made recognition of the same cells easy via CLSM (Figure 1(a)) and SEM (Figure 1(b)). Some shrinking during fixation and dehydration was detected, but the overall morphology of the cells was well preserved after fixation and processing for SEM. The GFP-HAS3 overexpressing cells were easily detached during processing (arrows in Figure 1), which indicates a decreased adhesion as a result of HAS overexpression, a finding in line with previous results [22].

### 3.2. Higher Resolution Reveals Tip Expansions of Protrusions and Dorsal Ruffling

**Figure 2:**
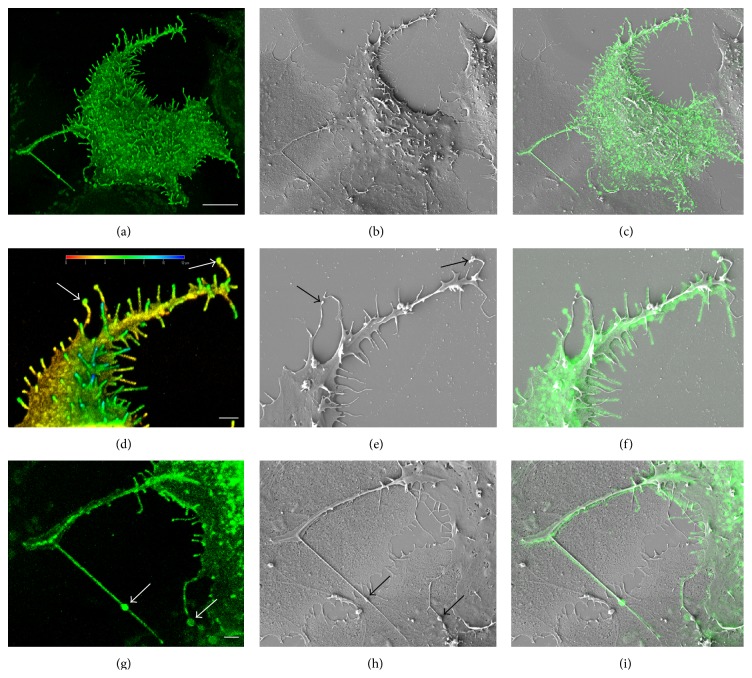
GFP-HAS3-induced plasma membrane protrusions imaged by CLEM. A GFP-HAS3 expressing MCF-7 cell imaged by 3D confocal microscopy in (a), (d), and (g) and by SEM in (b), (e), and (h). Merged images are presented in (c), (f), and (i). High magnification images from selected areas of the same cell presented in (a)–(c) are shown in (d)–(i). Arrows in (d), (e), (g), and (h) indicate the GFP-HAS3-positive bulbous expansions in both the body and tips of protrusions. Color depth coding was used to demonstrate the variable length of protrusions in (d). Scale bars 10 *μ*m in (a), 2 *μ*m in (d) and (g).

More detailed visualization of GFP-HAS3-positive MCF-7 cells was performed with higher magnification. A typical example of single GFP-HAS3-positive MCF-7 cell is shown in Figure 2. The morphology was relatively unchanged after sample processing for SEM (Figure 2) and GFP-HAS3-induced protrusions were preserved. As shown before, many of the HAS-induced protrusions have a dilated tip complex [14, 15], but it has been unclear if this dilation is an artefact resulting from high level of GFP-HAS3 fluorescence and light scattering. SEM confirmed that many of the HAS3-induced protrusions have a dilated tip complex (arrows in Figures 2(d) and 2(e)) and dilated areas are occasionally found also in the body of the protrusions (arrows in Figures 2(g) and 2(h)). The protrusions with dilated tips were similarly formed upon overexpression of HAS3 without GFP-tag (data not shown). Thickness of protrusions expressing high levels of GFP-HAS3 signal is overamplified in confocal microscopy because of light scattering. Protrusions also shrink and collapse during fixation and drying for SEM, which increases the differences in thickness observed between CLSM and SEM. As obtained by SEM, the HAS3-induced protrusions are extremely thin, typically between 70 and 130 nm in diameter.

**Figure 3:**
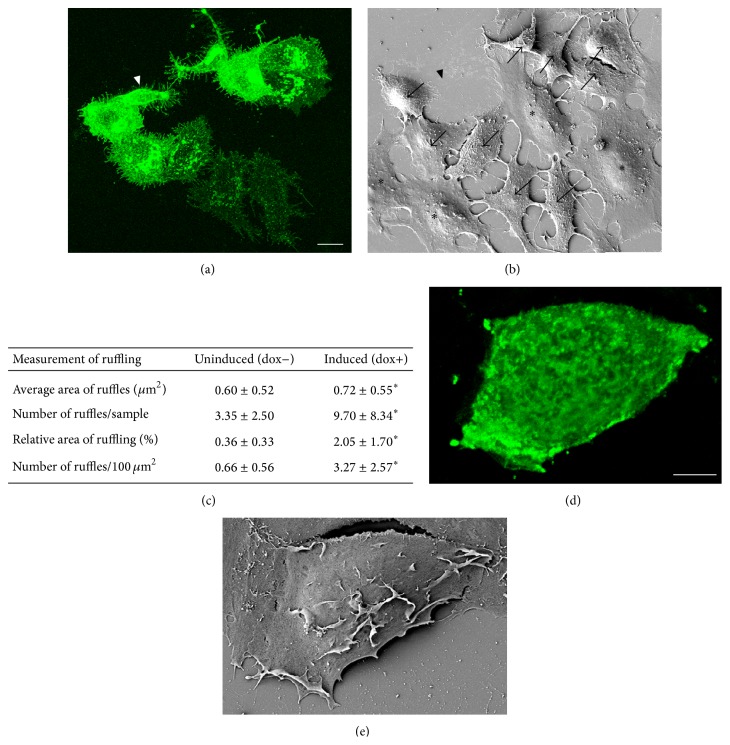
GFP-HAS3 expression induces plasma membrane ruffling. Confocal 3D projection of transiently transfected MCF-7 cells imaged by CLSM (a) and the corresponding area by SEM (b). Arrows in (b) indicate GFP-HAS3-positive cells with plasma membrane ruffling, while negative cells (asterisks) have smoother dorsal surface. Arrowheads in (a) and (b) indicate a cell that was lost during SEM processing. Stable, inducible transfections were utilized to quantify the dorsal ruffling of MCF-7 cells, which was significantly increased upon induction of GFP-HAS3. Significant difference (*P* < 0.05) in quantified parameters of ruffling is indicated by an asterisk (^*^) in table (c). *N* = 20 in both groups. A GFP-HAS3-positive cell treated with hyaluronidase before fixation is shown with CLSM and SEM in (d) and (e), respectively. Scale bars 10 *μ*m in (a) and 5 *μ*m in (d).

A typical morphology of cells with GFP-HAS3 overexpression was spindle-shaped, with no clear, single lamellipodia or distinguishable “front and rear” (arrows in Figure 3). Another typical feature of GFP-HAS3-positive cells was ruffling of the plasma membrane, appearing mainly on the apical faces of the plasma membrane. Comparison of negative cells (asterisks in Figures 3(a) and 3(b)) and GFP-HAS3-positive cells (arrows in Figures 3(a) and 3(b)) in transiently transfected cultures suggested that overexpression of HAS3 induces dorsal ruffling of the plasma membrane. To control if the ruffles are sensitive to hyaluronidase treatment, samples were treated with* streptomyces* hyaluronidase (5 TRU/mL, 30 min at 37°C) prior to fixation. The results showed that removal of hyaluronan did not completely destroy them (Figures 3(d) and 3(e)). This indicates that their structure is not completely dependent on pericellular hyaluronan.

### 3.3. Quantification of GFP-HAS3-Induced Plasma Membrane Ruffling

To confirm the findings obtained with transiently transfected cells, the dorsal plasma membrane ruffling was quantified utilizing the stable, inducible MCF-7 cell line expressing GFP-HAS3 [22]. The quantification of these complex structures of variable shape and size would be more time consuming by light microscopy, requiring relatively high magnification and stacks of confocal images. Thus SEM images were utilized for these measurements. The results showed that GFP-HAS3 expression significantly induced both the area and the amount of dorsal plasma membrane ruffling as compared to noninduced cells (Figure 3(c)).

### 3.4. Ultrastructure of Ruffles

**Figure 4:**
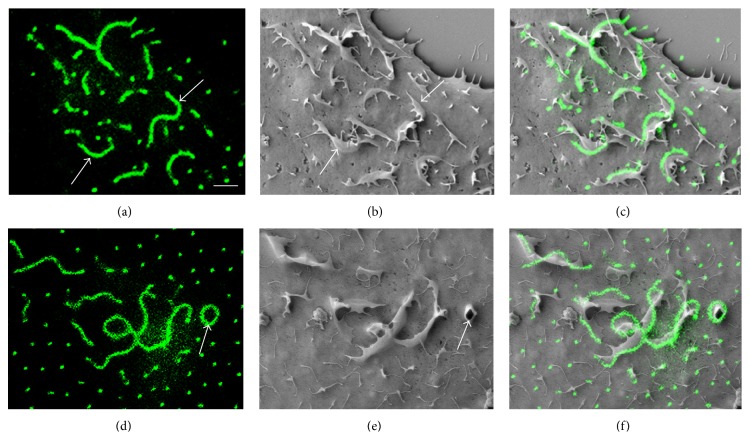
Ultrastructure of GFP-HAS3-positive dorsal ruffles. 2D confocal images showing the dorsal GFP-HAS3 signal are shown in (a) and (d) and corresponding SEM images in (b) and (e). Merged images are shown in (c) and (f), respectively. Many of the dorsal ruffles were linear or curved in shape (a) and provided a basis for several thinner protrusions (arrows in (b)). Occasionally, circular ruffles were detected (arrows in (d)-(e)). Scale bar 2 *μ*m.

Next high resolution SEM images were utilized to analyze the detailed structure of the HAS3-induced ruffles. Most of the ruffles appeared on the dorsal surface of the cell (Figures 2(b), 3, and 4) rather than on peripheral areas. GFP-HAS3 signal was detected on the apical plasma membrane of cells and accumulated on the ruffles. Furthermore, SEM revealed that many of the HAS-positive protrusions were embedded in the ruffle, suggesting that ruffles provide a basis for thinner protrusions. There was typically 1–5 or even higher number of protrusions arising from a single ruffle (arrows in Figure 4(b)). This indicates specific modeling of plasma membrane dynamics and the underlying actin network by HAS activity. Most of the ruffles were linear or curved, sheet-like protrusions of variable size, but some circular structures were also found (arrows in Figures 4(a), 4(b), 4(d), and 4(e)), which in line with the suggested dynamic formation of ruffles followed by constriction into circular structures before disappearing [5].

## 4. Discussion

### 4.1. CLEM as a Novel Method to Study HAS-Induced Changes in Cell Morphology

In cell biology, multiple imaging methods are usually required to solve a specific scientific problem. However, each imaging technique has its own limitations and separately does not fully answer the specific questions. Since the discovery of HAS3-induced plasma membrane extensions [11, 12, 23], hyaluronan-dependent plasma membrane modifications have been a specific cell biological question waiting for clarification. To solve this question, a straightforward CLEM protocol was developed to combine fluorescent and electron microscopic information of a single cell by using CLSM and SEM. The gridded coverslips were effective in relocating cells quickly and reliably over large areas but also allowed to study the detailed morphology of the plasma membrane. This correlative method is an inexpensive and simple way to image live or fixed cells with confocal microscopy prior to viewing them at the electron microscope level. The method can be performed without specific equipment and can be reliably utilized to answer many questions related to detailed localization of proteins and morphology of cultured cells.

### 4.2. Tip Complexes of Protrusions

We have shown before that many of the HAS-induced protrusions have a dilated tip complex [14, 15], but it has been unclear so far if this dilation is an artefact resulting from accumulation of GFP-HAS3 signal and light scattering. In this work, the existence of GFP-HAS3-positive bulbous expansions was confirmed in both the body and tips of the protrusions. Tip complexes act as putative sites of origin for shedding of hyaluronan-coated extracellular vesicles, which are potential carriers of hyaluronan and other active molecules [14, 15]. Additionally, tip complexes of protrusions are putative enrichment sites for specific proteins and act as functional areas for glucose uptake [24], which may be crucial for increased needs of glucose for hyaluronan synthesis. Furthermore, growth of protrusions [14, 15] and vesicle shedding [14, 15] are dependent on glucose supply, which makes the future studies of these tip complexes and their role in hyaluronan metabolism especially interesting.

### 4.3. Hyaluronan and Plasma Membrane Ruffling

Localization of hyaluronan and its receptors into ruffles has been reported in many different cell types, like fibroblasts [25], EGF-induced rat keratinocytes [26], CHO cells [4], and HaCaT cells [20]. Recently, also HAS3 localization into ruffles has been reported [20], but nobody has shown before that activity of hyaluronan synthesis itself induces ruffling of the plasma membrane. In this study, a clear increase was seen in the area and amount of ruffling after induction of HAS3 expression. Interestingly, growth factors that induce HAS expression and hyaluronan secretion, like EGF [26] and PDGF [27], induce dorsal membrane ruffing [10, 28]. As shown in this work, plasma membrane ruffling together with thin protrusions is a putative mechanism for HAS to increase the plasma membrane area in order to enhance its own activity. Moreover, small GTPase rac, which is one of the targets of hyaluronan-CD44 signaling [29], is required for membrane ruffling [30]. Furthermore, one of the cofactors of hyaluronan, MMP2 [31], localizes to the tips of ruffles [32], suggesting their role in promoting the degradation of ECM and invasive potential of cells in collaboration with hyaluronan interactions. All of these observations support the hypothesis that hyaluronan synthesis and plasma membrane ruffling are linked together.

### 4.4. Putative Mechanisms for Hyaluronan-Associated Plasma Membrane Ruffling

As shown here and before, active hyaluronan synthesis regulates both finger-like protrusions, like filopodia and microvilli, and sheet-like protrusive structures such as lamellipodia and ruffles. As well as finger-like protrusions, ruffles are structures usually erecting vertically from the dorsal cell surface. Pericellular, hydrated hyaluronan coat may provide a pulling force and mechanical support for growth and maintenance of these nonadherent structures. Interestingly, disturbance of the ECM-integrin interactions induces plasma membrane ruffling [8]. These interactions may be impaired in our model, where excess ventral hyaluronan due to HAS overexpression results in weaker cell attachment to the substratum. Furthermore, dorsal ruffles are suggested to arise as a consequence of inefficient lamellipodia adhesion and impaired migration rate [8], and assembly of ruffles inhibits actin flow to lamellipodia [7]. These findings may explain why highly increased levels of HAS overexpression lead to decreased lamellipodia formation and impaired migration rate [33].

### 4.5. Hyaluronan-Dependent Protrusions as Potential Sites for Glucose Uptake

Insulin or high glucose induces plasma membrane ruffling with simultaneous recruitment of glucose transporters (GLUT) into the plasma membrane ruffles of muscle cells [34]. Furthermore, GLUT translocation and insulin-stimulated glucose uptake are dependent on cortical actin remodeling and membrane ruffling [35]. These results indicate that hyaluronan, plasma membrane dynamics, and glucose metabolism are linked to each other and suggest that ruffles act as potential sites for cellular glucose uptake. This hypothesis fits well with previous reports on positive correlation between cellular glucose levels and activity of hyaluronan synthesis [14, 15, 36, 37] and suggests that HAS directly or indirectly recruits glucose transporters into the plasma membrane ruffles and protrusion tip complexes to enhance its own activity.

### 4.6. Future Prospects

We have shown previously that active hyaluronan production induces formation of plasma membrane extensions [11, 12] and blebbing of extracellular vesicles [14, 15]. The induction of plasma membrane ruffles, the main finding of this work, strengthens the role of active hyaluronan synthesis as a regulator of plasma membrane dynamics, regulating cell behavior in health and disease. Future studies will settle in more detail the dynamics and regulation of HAS-induced ruffles and their relationship with hyaluronan secretion and other cell functions, like secretion of extracellular vesicles and regulation of glucose uptake.

## Abbreviations

CLSM: Confocal laser scanning microscopy
DIC: Differential interference contrast
ECM: Extracellular matrix
EGF: Epidermal growth factor
GFP: Green fluorescent protein
GLUT: Glucose transporter
HAS: Hyaluronan synthase
MMP: Matrix metalloproteinase
PDGF: Platelet-derived growth factor
SEM: Scanning electron microscopy.
